# Crosstalk of cuproptosis-related prognostic signature and competing endogenous RNAs regulation in hepatocellular carcinoma

**DOI:** 10.18632/aging.205273

**Published:** 2023-12-10

**Authors:** Jun Zhu, Jingyan Wang, Hong Liu, Tong Lei, Jiankang Yang, Sheng Lan, Haokun Jian, Hanlu Fang, Yu Zhang, Kuiwu Ren, Fei Zhong

**Affiliations:** 1 Department of Oncology, Guoyang County People’s Hospital, Guoyang Branch of Anhui Provincial Hospital, Guoyang 233607, Anhui, China; 2 Department of Oncology, Fuyang Hospital of Anhui Medical University, Fuyang 236000, Anhui, China; 3 Department of Anesthesia, Shaoxing People’s Hospital, Shaoxing 312000, Zhejiang, China; 4 Department of Cardiovascular Medicine, Fuyang Hospital of Anhui Medical University, Fuyang 236000, Anhui, China; 5 The First Affiliated Hospital, Nanchang University, Nanchang 330006, Jiangxi, China; 6 Department of Cardiac Surgery, The First Affiliated Hospital of Anhui Medical University, Hefei 230000, Anhui, China; 7 The Second Clinical College of Guangzhou Medical University, Guangzhou 510030, Guangdong, China; 8 School of Basic Medical Sciences, Xinxiang Medical University, Xinxiang 453003, Henan, China; 9 Institute of Medical and Health Science, Hebei Medical University, Shijiazhuang 050017, Hebei, China; 10 The Second Clinical Medical College, Lanzhou University, Lanzhou 730000, Gansu, China; 11 Department of Hepatobiliary Surgery, Fuyang People’s Hospital, Fuyang 236000, Anhui, China; 12 Department of Oncology, The First Affiliated Hospital of Anhui Medical University, Hefei 230000, Anhui, China

**Keywords:** cuproptosis, metabolism, signature, hepatocellular carcinoma, immune microenvironment

## Abstract

Background: Cuproptosis is a new type of programmed cell death involved in the regulation of neuroendocrine tumors, immune microenvironment, and substance metabolism. However, the role of cuproptosis-related genes (CRGs) in Hepatocellular carcinoma (HCC) remains unclear.

Method: Through multiple bioinformatics analysis, we constructed a prognostic gene model and competing endogenous RNA (ceRNA) network. The correlation between CRGs and prognosis, immune infiltration, immune checkpoints, microsatellite instability (MSI) and tumor mutational burden (TMB) was analyzed by Kaplan-Meier curve, univariate Cox, multivariate regression, and Spearman’s analysis in HCC patients. Besides, the qRT-PCR and immunohistochemistry assays were used to determine prognostic CRGs mRNA and protein expression in HCC.

Results: We established a novel 3-gene signature related to CRGs for evaluating the prognosis of HCC patients. HCC patients with high risk scores had a poor prognosis with an area under the curve of 0.737, 0.646, and 0.634 on 1-year, 3-year, and 5-year receiver operating characteristic curves. Significant correlation was observed between prognostic CRGs and immune infiltration, immune checkpoints, MSI and TMB. We also developed five ceRNA networks to regulate the occurrence and progression of HCC. CDKN2A, DLAT, and PDHA1 protein expression was up-regulated in HCC versus normal tissues. Besides, the mRNA expression levels of CDKN2A, DLAT, GLS, and PDHA1 were elevated in the HCC cell lines compared to the normal liver cell lines.

Conclusions: This novel prognostic CRGs signature could be accurately predict the prognosis of patients with HCC. The ceRNA regulatory network might be potential prognostic biomarkers and therapeutic targets for HCC patients.

## INTRODUCTION

Liver cancer is the sixth most common cancer and the third leading cause of cancer death [1]. Hepatocellular carcinoma (HCC), accounting for 75-85% of primary liver cancer, is closely associated with drinking, viral hepatitis, aflatoxin, and genetics [2]. Resection, transplantation, and ablation are treatments for HCC. However, due to tumor heterogeneity, the prognosis of patients with HCC is unsatisfactory, with a 5-year survival rate of only 18 percent [3]. Although liver transplantation is currently the preferred treatment for patients with early-stage HCC, most HCC patients with advanced disease are often not candidates for liver transplantation because the cancer has spread throughout the body or because the liver function is poor [4]. Although many biomarkers have been identified to predict the prognosis of HCC patients, they are still in the molecular research stage and are not yet widely used in clinical applications [5]. Therefore, it is of great significance to establish more effective prognostic models and improve therapeutic strategies for HCC.

Copper is an essential trace element for the human body and an important cofactor for all organisms [6]. Maintaining the balance of copper content in organisms is essential for the survival and development of organisms [7]. Cuproptosis is a novel cell death mechanism, which occurs by the direct binding of copper to the fatty acylated components of the tricarboxylic acid (TCA) cycle to induce the aggregation of fatty acylated proteins and the destabilization of Fe-S cluster proteins, resulting in protein toxic stress and ultimately cell death [8]. There is increasing evidence that cuproptosis has a large impact on the prognosis of tumors, such as clear cell renal cell carcinoma [9] and melanoma [10]. Competing endogenous RNA (ceRNA) networks are considered to play a critical role in many types of cancer, and the novel potential ceRNA axis of cuproptosis-related genes (CRGs) were identified in uterine corpus endometrial carcinoma [11] and breast cancer [12] to regulate cancer progression. It is reported that dysregulation of copper homeostasis is associated with poor outcomes in HCC patients [13]. The prognostic value and potential ceRNA network of CRGs in HCC has not been elucidated.

In this study, based on the identified 12 CRGs, we comprehensively analyzed the expression level of CRGs in HCC and constructed a signature to predict the survival outcomes of HCC patients and the ceRNA regulatory network in HCC. This provides new ideas for finding therapeutic targets and prognostic biomarkers for HCC.

## MATERIALS AND METHODS

### Data collection and preprocessing

The Cancer Genome Atlas (TCGA) (https://www.cancer.gov/tcga) database was employed to download RNA-sequencing expression profiles and corresponding clinical information for HCC. The clinical information of patients with HCC is presented in Table 1. All data analysis was performed with the R (version 4.0.5) and R Bioconductor packages.

**Table 1:** The clinical information of HCC patients in TCGA.

	**Clinical characters**	**Number**
Gender	Male	250
	Female	121
Status	Alive	241
	Dead	130
Age	Mean (SD)	59.4 (13.5)
	Median [MIN, MAX]	61 [16,90]
TNM stage	I	171
	II	86
	III	85
	IV	5
T stage	T1	181
	T2	94
	T3	80
	T4	13
	TX	1
N stage	N0	252
	N1	4
	NX	114
M stage	M0	266
	M1	4
	MX	101
Grade	G1	55
	G2	177
	G3	122
	G4	12

### Identification of differentially expressed cuproptosis-related genes

According to previous studies, a total of 12 CRGs were obtained, including FDX1, LIAS, LIPT1, DLD, DLAT, PDHA1, PDHB, MTF1, GLS, CDKN2A, SLC31A1, and ATP7B [8]. The difference in CRGs expression in HCC and normal tissues was identified using the “limma” and “reshape2” packages. Then, we used the Search Tool for The Retrieval of Interacting Genes (STRING) (http://string-db.org/) to establish the protein-protein interaction (PPI) network of CRGs.

### Functional enrichment analysis of CRGs

Metascape (http://metascape.org) was used to perform Gene Ontology (GO) and Kyoto Encyclopedia of Genes and Genomes (KEGG) analysis. (Settings: Min Overlap: 3; *P*-Value Cutoff: 0.01; Min Enrichment: 1.5).

### Prognostic analysis of CRGs and establishment of prognostic model

The K-M survival analysis with log-rank test were used to compare the survival difference between high-expression and low-expression groups to identify potential prognostic biomarkers. The *P*-values and hazard ratios (HRs) with 95% confidence intervals (CIs) were obtained by log-rank test. Based on these potential CRGs, LASSO Cox regression analysis was applied to construct a cuproptosis-related prognostic model. After calculating the risk score as follows: Risk score = (0.1584)*CDKN2A+(0.2594)*DLAT+(0.1042)*GLS, HCC patients were divided into two parts (low- and high-risk groups) according to the median risk score, and the overall survival (OS) time was drawn using Kaplan–Meier method. The receiver operating characteristic (ROC) curve was drawn using the “time-ROC” R packages to evaluate the performance of the risk model. The forest is plotted by the “forest plot” R package showing the *P*-value, HR and 95% CI for each variable. Considering the clinical characteristics and prognostic signature, a predictive nomogram was constructed to predict the overall survival of HCC patients at 1, 3 and 5 years.

### Immune infiltration, TMB, MSI analysis

The Tumor Immune Estimation Resource, version 2 (TIMER2.0, http://timer.cistrome.org/) database is a comprehensive resource for systematic evaluation of immune infiltration of various cancer types [14]. The “Gene” module of TIMER was used to investigate the relationship between prognostic CRGs and immune infiltration level. In TMB and MSI analysis, Spearman’s correlation analysis was performed to calculate the correlation between prognostic CRGs and TMB and MSI score.

### Construction of ceRNA network

We explored the relationship between prognostic CRGs and clinical stage of HCC using the Gene Expression Profiling Interactive Analysis (GEPIA) (http://gepia2.cancer-pku.cn/#index) database. Then, miRTarbase (https://mirtarbase.cuhk.edu.cn/~miRTarBase/miRTarBase_2022/php/index.php) and Tarbase v.8 (https://dianalab.e-ce.uth.gr/html/diana/web/index.php?r=tarbasev8/index) were utilized to explore potential miRNA targets bound to CRGs. Based on the identified miRNAs, potential upstream lncRNAs associated with miRNAs were further detected using miRNet (https://www.miRNet.ca/). In addition, the expression and prognostic value of these miRNA and lncRNA targets were further explored with the help of the StarBase (http://starbase.sysu.edu.cn/), and the correlation analysis of mRNA-miRNA, mRNA-lncRNA, and miRNA-lncRNA was performed.

### Cell culture

The normal hepatic cell (LO2) and HCC cell lines (7721 and HepG2) were purchased from Procell Biotech (Wuhan, China). The cell culture conditions were DMEM with 10% fetal bovine serum (VivaCell, Shanghai, China), and 5% CO_2_, 37° C.

### Quantitative real-time polymerase chain reaction (qRT-PCR)

RNA was extracted by the TRIzol reagent, and cDNA was obtained from the reverse transcription using PrimeScript™ kit. Quantitative expression was detected using SYBR Green qPCR Mix. Primer sequences of all target genes and internal control are shown as follows: CDKN2A forward:5′-GATCCAGGTGGGTAGAAGGTC-3′ and CDKN2A reverse: 5′-CCCCTGCAAACTTCGTCCT-3′; DLAT forward: 5′-CGGAACTCCACGAGTGACC-3′ and DLAT reverse: 5′-CCCCGCCATACCCTGTAGT-3′; GLS forward: 5′-AGGGTCTGTTACCTAGCTTGG-3′ and GLS reverse: 5′-ACGTTCGCAATCCTGTAGATTT-3′; PDHA1 forward: 5′-TGGTAGCATCCCGTAATTTTGC-3′ and PDHA1 reverse: 5′-ATTCGGCGTACAGTCTGCATC-3′; The data was calculated as 2^−ΔΔCt^.

### Immunohistochemistry analysis

The Human Protein Atlas (HPA) (www.proteinatlas.org/) database includes immunohistochemistry data for common cancer types [15]. The database was used to detect the protein expression of CRGs in normal and tumor tissues in the “tissue” and “pathology” modules.

### Statistical analysis

The predictive value of the CRGs signature was assessed by Cox regression and Lasso regression analyses. The Kaplan–Meier method was used to evaluate the survival of HCC patients. The AUC was calculated from the ROC curve to evaluate the accuracy of the prediction. Spearman’s analysis was used to investigate the relationship between CRGs expression and immune infiltration, TMB and MSI score. Statistical significance was defined as *P* < 0.05.

### Availability of data and materials

The datasets presented in this study can be found in online repositories. The names of the repository/repositories and accession number(s) can be found in the article/Supplementary Material.

## RESULTS

### Expression of CRGs in HCC

**Figure 1:**
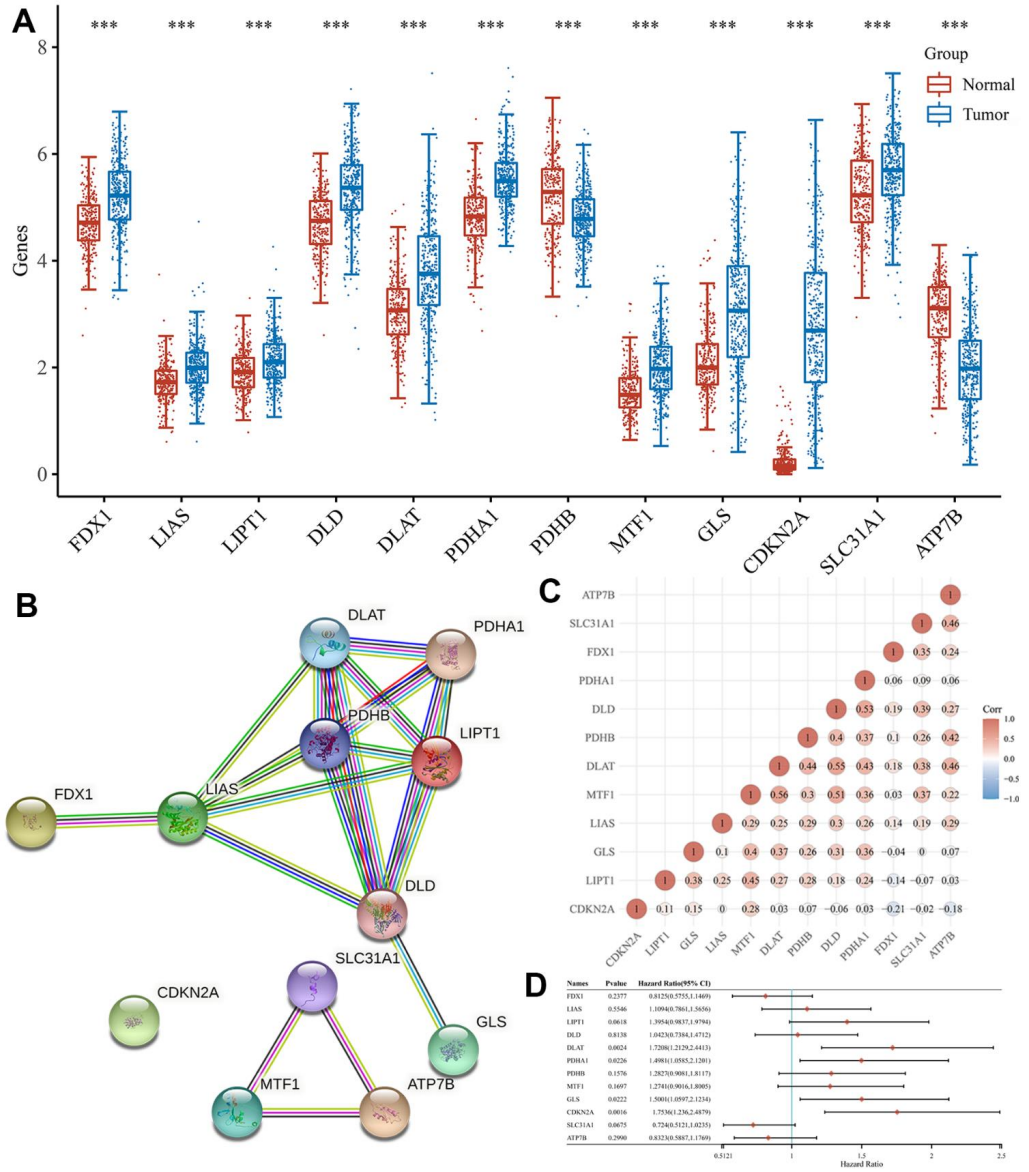
**Expression and interaction of CRGs.** (**A**) The expression of 12 CRGs in HCC and normal tissues. Tumour, red. Normal, blue. (**B**) PPI network of 12 CRGs. (**C**) The correlation among 12 CRGs. (**D**) The forest plot shows the prognosis of CRGs in HCC.

The expression patterns of 12 CRGs in HCC were analyzed using TCGA and The Genotype-Tissue Expression (GTEx) databases, and the results showed that all 12 genes were differentially expressed between HCC and normal tissues (Figure 1A). Specifically, the expression of FDX1, LIAS, LIPT1, DLD, DLAT, PDHA1, MTF1, GLS, CDKN2A, SLC31A1 and ATP7B was upregulated in HCC tissues versus normal tissues, while the expression of PDHB was downregulated (Figure 1A, all *P* < 0.05). Based on the STRING database, the PPI network revealed that LIAS, DLAT, PDHA1, PDHB, LIPT1, DLD were the hub genes (Figure 1B). Correlation analysis to determine the interaction between these genes indicated that most genes were positively correlated with each other (Figure 1C).

### Functional enrichment analysis of CRGs

To clarify the role of CRGs, we conducted GO and KEGG analysis by Metascape (Table 2). GO molecular function analysis revealed that 12 CRGs were mainly associated with pyruvate dehydrogenase activity and oxidoreductase activity. GO Cellular Components analysis suggested that 12 CRGs were mainly involved in mitochondrial pyruvate dehydrogenase complex and mitochondrial matrix. We also found that these 12 CRGs were mainly involved in acetyl-CoA biosynthetic process from pyruvate, protein maturation and ion homeostasis in GO Biological Processes analysis. KEGG pathway analysis revealed that 12 CRGs were enriched in Citrate cycle (TCA cycle), Platinum drug resistance and Biosynthesis of cofactors.

**Table 2:** Significantly GO and KEGG analysis with metascape.

**Term**	**Category**	**Description**	**Count**
GO:0004738	GO Molecular Functions	pyruvate dehydrogenase activity	4
GO:0016491	GO Molecular Functions	oxidoreductase activity	5
GO:0006086	GO Biological Processes	acetyl-CoA biosynthetic process from pyruvate	4
GO:0051604	GO Biological Processes	protein maturation	3
GO:0050801	GO Biological Processes	ion homeostasis	3
GO:0005967	GO Cellular Components	mitochondrial pyruvate dehydrogenase complex	4
GO:0005759	GO Cellular Components	mitochondrial matrix	8
hsa00020	KEGG Pathway	Citrate cycle (TCA cycle)	4
hsa01524	KEGG Pathway	Platinum drug resistance	3
hsa01240	KEGG Pathway	Biosynthesis of cofactors	3

### Construction of a cuproptosis-related prognostic gene model

**Figure 2:**
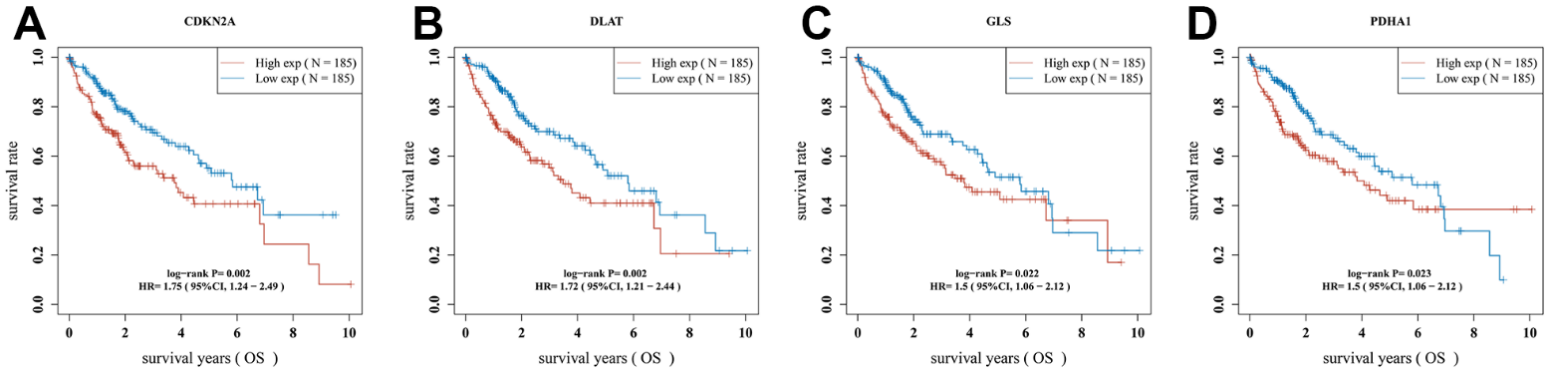
**The overall survival curve of CRGs in HCC patients in the high-/low-expression group.** (**A**) CDKN2A (**B**) DLAT (**C**) GLS (**D**) PDHA1.

**Figure 3:**
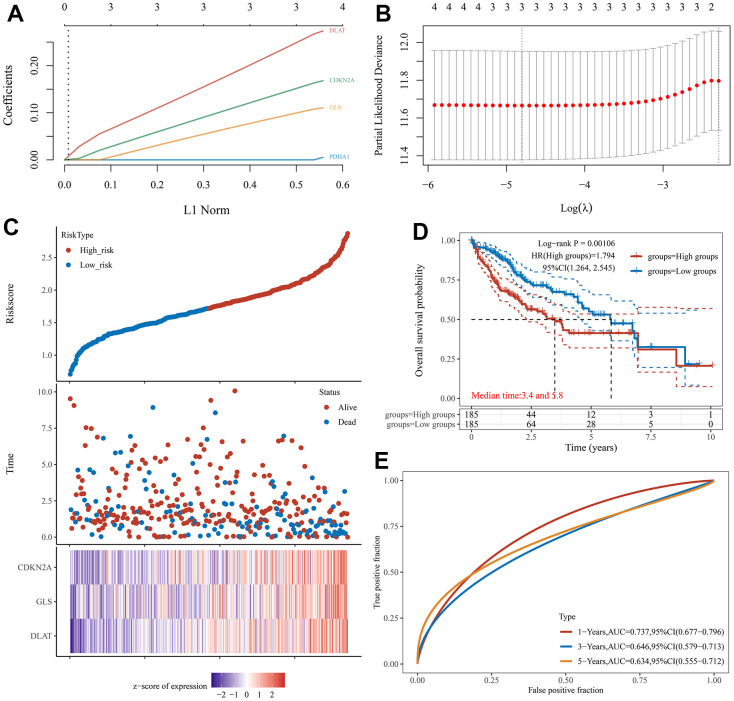
**Construction of a prognostic CRGs model in HCC.** (**A**, **B**) Coefficient and partial likelihood deviance of prognostic model. (**C**) Distribution of risk score, survival status, and the expression of 3 prognostic CRGs in HCC. (**D**) Survival curve of HCC patients with high/low-risk score. (**E**) The AUC time-dependent ROC curves in 1-, 3-, and 5-year.

Univariate Cox regression analysis was used to screen a potential cuproptosis-related prognostic marker for HCC. The results showed that a total of 4 genes with prognostic value were identified (Figure 1D). Kaplan-Meier survival curves indicated that a poor survival rate in HCC patients with high expression of CDKN2A (Figure 2A, *P* = 0.002), DLAT (Figure 2B, *P* = 0.002), GLS (Figure 2C, *P* = 0.022) and PDHA1 (Figure 2D, *P* = 0.023). Based on these four prognostic genes, LASSO Cox regression analysis was performed to construct a prognostic gene model (Figure 3A, 3B). HCC patients were divided into low-risk and high-risk groups according to the risk score, and more HCC patients were found to be alive in the high-risk group versus the low-risk group (Figure 3C). In addition, the high-risk group showed a lower overall survival rate than the low-risk group (Figure 3D, *P* = 0.00106, HR = 1.794), with AUCs of 0.737, 0.646, and 0.634 in the 1-year, 3-year, and 5-year ROC curves, respectively (Figure 3E).

### Establishment of a predictive nomogram

**Figure 4:**
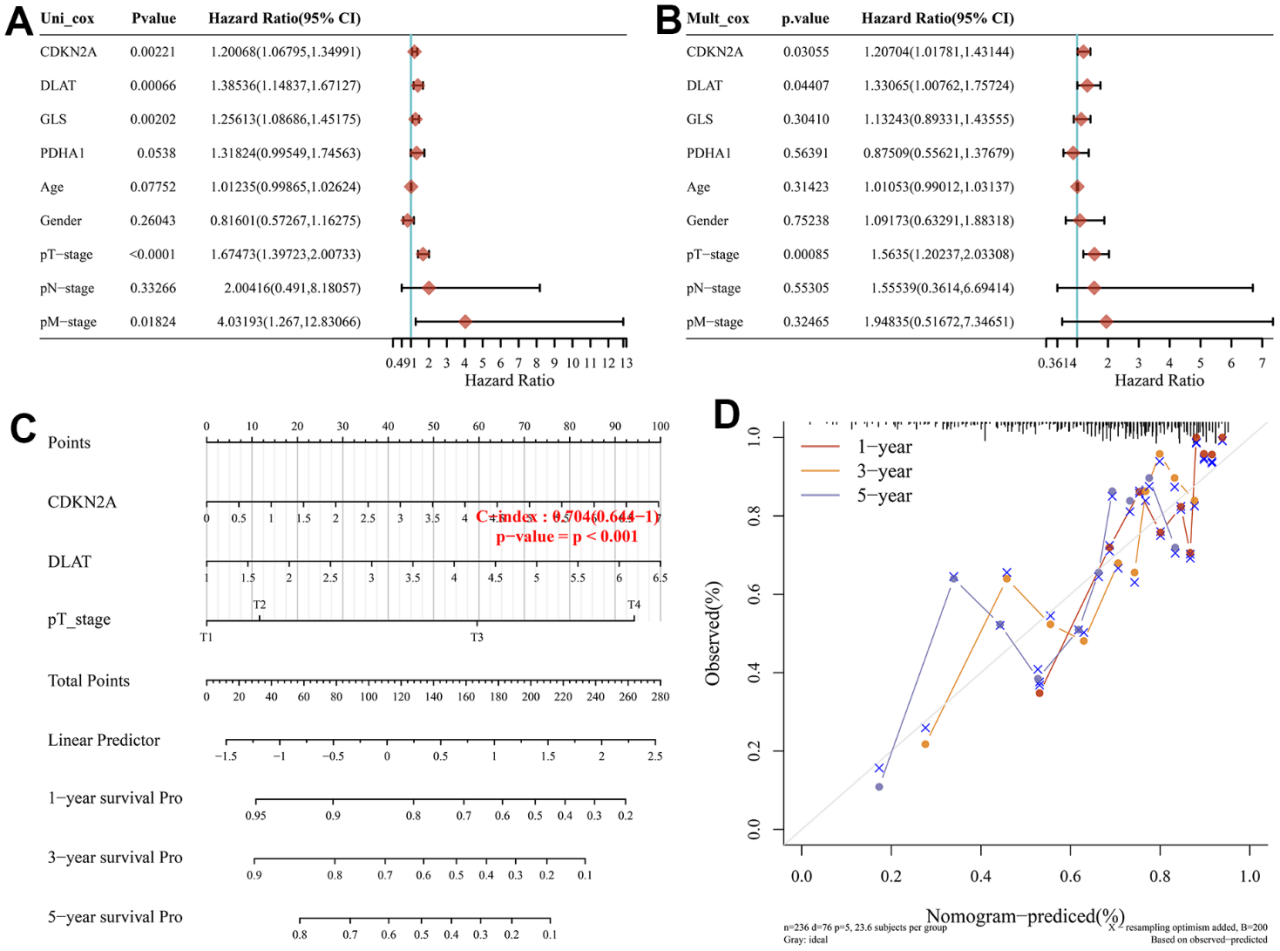
**Construction of a predictive nomogram.** (**A**, **B**) Univariate and multivariate Cox regression considering clinical the parameters and prognostic CRGs in HCC. (**C**) Development of a Nomogram to predict the 1-year, 3-year, and 5-year survival probability of HCC patients. (**D**) The calibration curve was to verify the efficacy of nomogram.

Considering these prognostic CRGs and the clinicopathological characteristics, we have performed univariate and multifactorial analyses to further construct predictive nomogram to estimate the probability of survival. The results indicated that CDKN2A, DLAT, and pT staging were independent factors affecting the prognosis of HCC patients (Figure 4A, 4B). We then constructed a clinical predictive nomogram based on CDKN2A, DLAT, pT staging, which showed excellent predictive performance compared to the ideal model for 3-year and 5-year survival probability (Figure 4C, 4D).

### CRGs are associated with tumor immune processes in HCC

**Figure 5:**
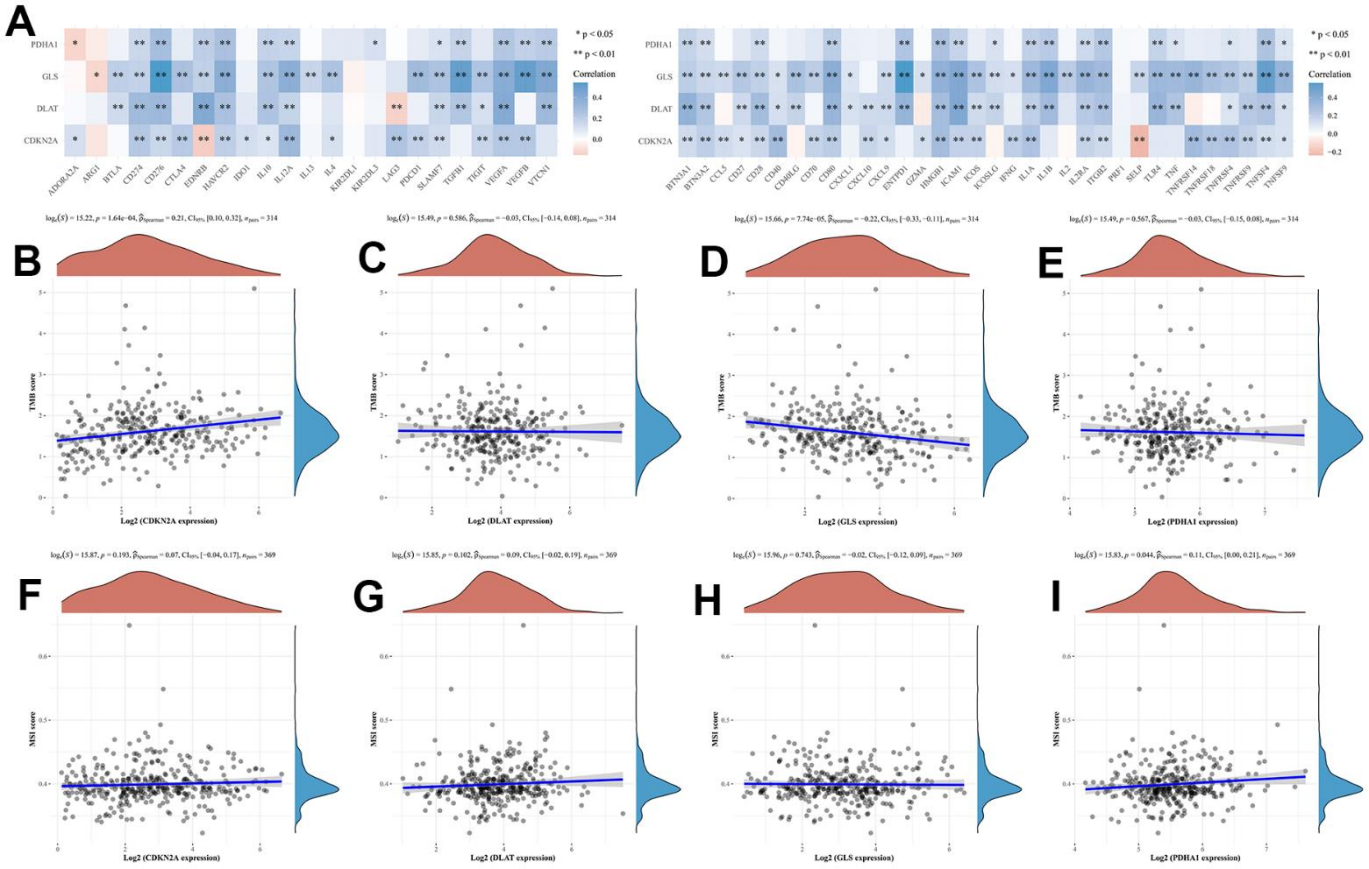
**The relationship between CRGs and immune checkpoints, TMB and MSI in HCC patients.** (**A**) immune checkpoints (**B**–**E**) TMB. TMB, tumour mutation burden. (**F**–**I**) MSI. microsatellite instability.

**Figure 6:**
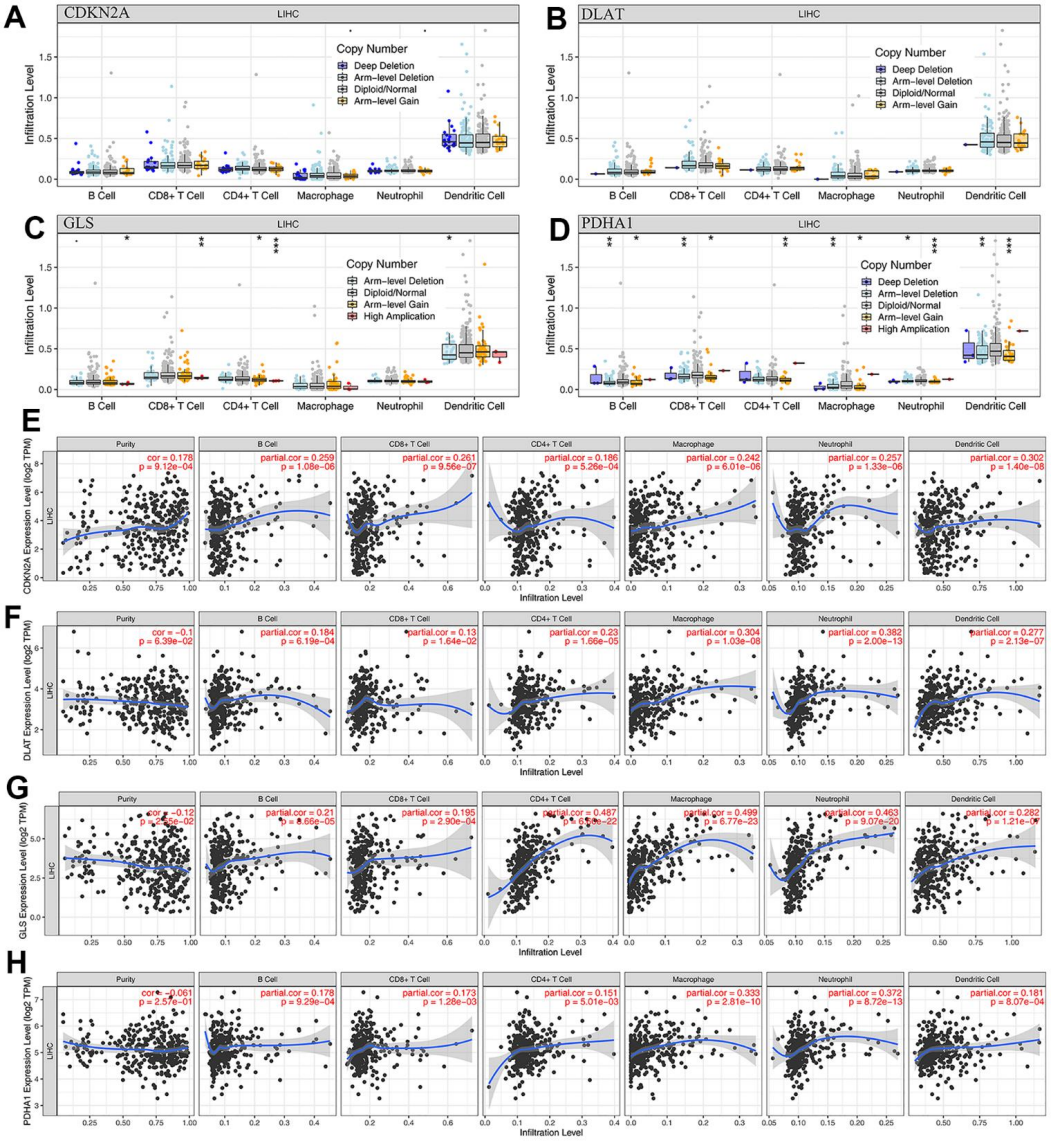
**The relationship of immune cell infiltration with prognostic CRGs level in HCC.** (**A**–**D**) The infiltration level of various immune cells under different copy numbers of 4 prognostic CRGs in HCC. (**E**–**H**) The correlation of the abundance of immune cells and the expression of 4 prognostic CRGs in HCC.

In this study, we analyzed the correlation between the expression levels of prognostic CRGs (CDKN2A, DLAT, GLS, and PDHA1) and immune processes in HCC. We found that these 4 prognostic CRGs were positively associated with most immunoinhibitors and immunostimulators (Figure 5A). TMB and MSI may serve as predictive biomarkers for cancer immunotherapy [16, 17]. We investigated the correlation between CRGs and TMB as well as MSI in HCC. The results showed that TMB scores were positively correlated with CDNK2A (Figure 5B, *P* = 1.64e-04) expression and negatively correlated with GLS (Figure 5D, *P* = 7.74e-05) expression, but not with DLAT (Figure 5C, *P* = 0.586) and PDHA1 (Figure 5E, *P* = 0.567) expression. In MSI analysis, MSI scores of HCC patients increased as PDHA1 (Figure 5I, *P* = 0.044) expression increased. However, MSI score was not significantly correlated with the expression of CDNK2A (Figure 5F, *P* = 0.193), DLAT (Figure 5G, *P* = 0.102), and GLS (Figure 5H, *P* = 0.743). In the immune infiltration analysis, we found that there were no significant changes in the level of immune cell infiltration with different CDNK2A and DLAT copy numbers in HCC (Figure 6A, 6B). Different GLS and PDHA1 copy numbers were significantly correlated with the level of partial immune cell infiltration (Figure 6C, 6D). Moreover, the results revealed that the expression of CDKN2A, DLAT, GLS, and PDHA1 positively correlated with the abundance of B cells, CD8+ T cells, CD4+ T cells, macrophages, neutrophils, and dendritic cells (Figure 6E–6H).

### Development of LncRNA-miRNA-mRNA network

**Figure 7:**
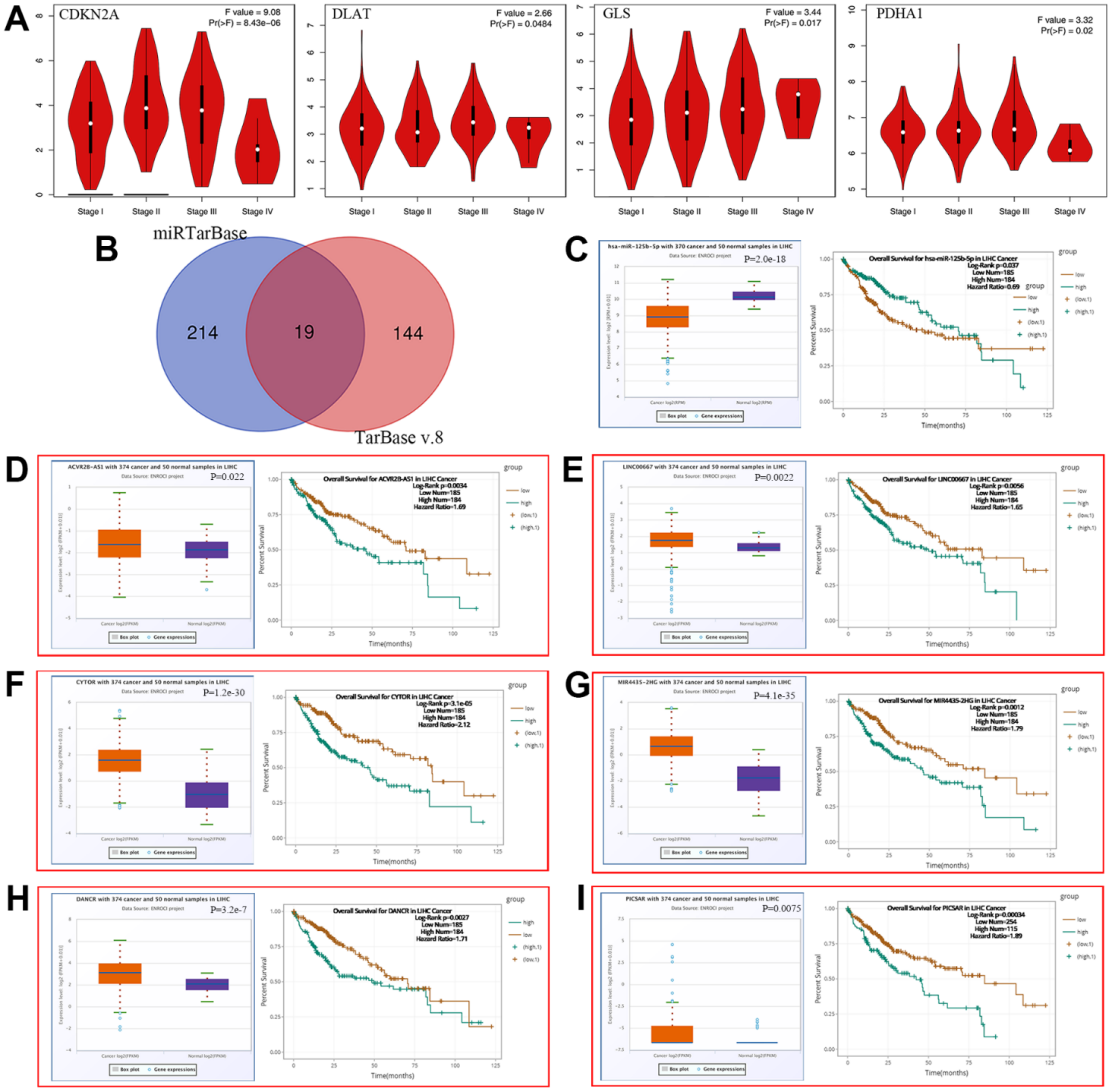
**Construction of ceRNA network in HCC.** (**A**) The relationship between 4 prognostic CRGs and clinical stage. (**B**) A Results of miRNA target predicted by mirTarBase and TarBase V.8. (**C**–**I**) The overall survival and expression of (**C**) hsa-miR-125b-5p (**D**) ACVR2B-AS1 (**E**) LINC00667 (**F**) CYTOR (**G**) MIR4435-2HG (**H**) DANCR (**I**) PICSAR in HCC.

We also elucidated the correlation between prognostic CRGs and clinical staging (Figure 7A), showing that CDKN2A (*P* = 8.43e-06), DLAT (*P* = 0.0484), GLS (*P* = 0.017), and PDHA1 (*P* = 0.02) were associated with clinical staging. To elucidate the potential molecular mechanisms of these four genes in HCC, we constructed a LncRNA-miRNA-mRNA network. According to the predicted results of mirTarBase and TarBase V.8, a total of 19 miRNAs (40 miRNA-mRNA pairs) were identified as potential miRNA targets for these four genes (Figure 7B and Supplementary Table 1). The ceRNA-based hypothesis suggested that the expression of upstream miRNAs should negatively regulate mRNA. Further analysis revealed that 8 miRNAs (Supplementary Figure 1A and Figure 7C) were expressed down-regulated in HCC. And the prognostic analysis showed that only HCC patients with high levels of hsa-miR-125b-5p (the upstream miRNA of GLS and CDKN2A) (Figure 7C, *P* = 2.0e-18) had better overall survival (Figure 7C, *P* = 0.037). Moreover, we explored hsa-miR-125b-5p upstream lncRNAs using the miRNet database, obtaining 47 miRNA-lncRNA pairs (Supplementary Table 2). Under the ceRNA hypothesis, lncRNAs should negatively regulate miRNAs as well as positively regulate mRNA. We analyzed the expression and prognosis of these lncRNAs in HCC and revealed that 34 LncRNAs were expressed upregulated in HCC (Supplementary Figure 1 and Figure 7D–7I), while only ACVR2B-AS1 (Figure 7D, *P* = 0.022), LINC00667 (Figure 7E, *P* = 0.0022), CYTOR (Figure 7F, *P* = 1.2e-30), MIR4435-2HG (Figure 7G, *P* = 4.1e-35), DANCR (Figure 7H, *P* = 3.2e-7), and PICSAR (Figure 7I, *P* = 0.0075) that were up-regulated in HCC and linked to poor prognosis (Figure 7D, *P* = 0.0034, Figure 7E, *P* = 0.0056, Figure 7F, *P* = 3.1e-05, Figure 7G, *P* = 0.0012, Figure 7H, *P* = 0.0027, Figure 7I, *P* = 0.00034). We further investigated the correlation between mRNA-miRNA, miRNA-lncRNA and mRNA-lncRNA pairs (Table 3). All pairs were consistent with the ceRNA mechanism except for the hsa-miR-125b-5p-ACVR2B-AS1, hsa-miR-125b-5p-LINC00667, hsa-miR-125b-5p-PICSAR, and GLS-DANCR pairs. Considering these three levels, five LncRNA-miRNA-mRNA (CYTOR-hsa-miR-125b-5p-CDKN2A, MIR4435-2HG-hsa-miR-125b-5p-CDKN2A, DANCR-hsa-miR-125b-5p-CDKN2A, CYTOR-hsa-miR-125b-5p-GLS, MIR4435-2HG-hsa-miR-125b-5p-GLS) axes were constructed and had significant prognostic value in HCC.

**Table 3:** Correlation analysis between mRNA-miRNA, miRNA-lncRNA and mRNA-lncRNA pairs.

**miRNA**	**mRNA**	**R**	***P*-value**
hsa-miR-125b-5p	CDKN2A	-0.132	1.09E-02*a
hsa-miR-125b-5p	GLS	-0.265	2.26E-07***a
miRNA	lncRNA		
hsa-miR-125b-5p	CYTOR	-0.372	1.35E-13***a
hsa-miR-125b-5p	MIR4435-2HG	-0.307	1.70E-09***a
hsa-miR-125b-5p	ACVR2B-AS1	0.052	3.18E-01
hsa-miR-125b-5p	DANCR	-0.26	3.98E-07***a
hsa-miR-125b-5p	LINC00667	0.125	1.64E-02*a
hsa-miR-125b-5p	PICSAR	-0.055	2.92E-01
mRNA	lncRNA		
CDKN2A	CYTOR	0.287	1.58E-08***a
CDKN2A	MIR4435-2HG	0.199	1.10E-04***a
CDKN2A	DANCR	0.118	2.22E-02*a
GLS	CYTOR	0.261	3.11E-07***a
GLS	MIR4435-2HG	0.321	2.20E-10***a
GLS	DANCR	0.092	7.57E-02

^a^These results are statistically significant.

*P-value < 0.05; **P-value < 0.01; ***P-value <0.001.

### Verification of the mRNA and protein expression of genes

**Figure 8:**
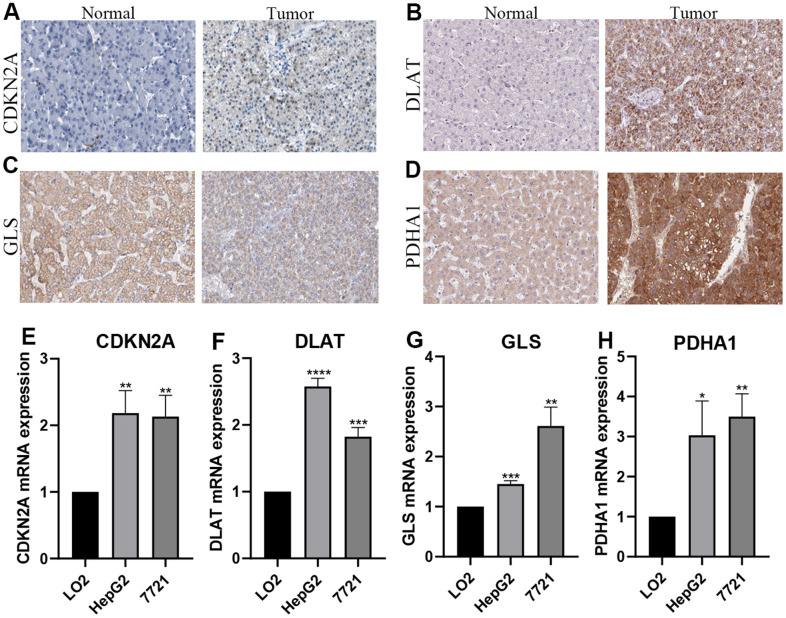
**Verification of the mRNA and protein expression.** (**A**–**D**) Images from the Human Protein Atlas show the protein expression of 4 prognostic CRGs in HCC and corresponding normal tissues. (**E**–**H**) qRT-PCR analysis detected the mRNA expression 4 prognostic CRGs in normal cell line (Lo2), and HCC cell lines (7721, HepG2). *P < 0.05; **P < 0.01; ***P < 0.001.

Immunohistochemical images of CDKN2A, DLAT, GLS, and PDHA1 were obtained from the HPA database. Compared with adjacent non-tumor tissues, CDKN2A, DLAT, and PDHA1 protein expression was upregulated in HCC tissues. But there was no significant difference in the protein level of GLS between HCC and adjacent non-tumor tissues (Figure 8A–8D). The expression of these proteins were predominately located in Cytoplasmic/membranous. The mRNA expression levels of CDKN2A, DLAT, GLS, and PDHA1 were elevated in the HCC cell lines (7791 and HepG2) compared to the normal liver cell line (LO2) (Figure 8E–8H).

## DISCUSSION

Cuproptosis is a new copper-dependent cell death method first proposed in cell this year. Copper directly binds to fatty acylated moieties of the tricarboxylic acid cycle to form aggregates, resulting in cytotoxicity and even cell death. Compared with normal tissue, copper has been shown to accumulate in the serum of patients with cirrhosis [18] or more cancers [19, 20]. Copper ions provide metal nutrients required for angiogenesis of tumor growth and metastasis by participating in redox, mitochondrial energy metabolism, aerobic glycolysis, and various enzymatic pathways. Copper import genes (SL31A1) and copper export genes (ATP7A and ATP7B), which are responsible for regulating intracellular copper concentration, work together to maintain intracellular copper homeostasis. When this homeostasis is out of balance, it leads to an increased incidence of HCC and promotes malignant progression [21]. Due to the prominent role of copper death, it is necessary to explore novel copper death-related biomarkers and establish an accurate cuproptosis-related HCC prognostic model.

We first examined the expression of 12 CRGs in HCC. Compared with normal tissues, FDX1, LIAS, LIPT1, DLD, DLAT, PDHA1, MTF1, GLS, CDKN2A and SLC31A1 were up-regulated in HCC, whereas PDHB and ATP7B were down-regulated. These results are consistent with those of a previous study that created a prognostic lncRNA profile associated with cuproptosis to predict response to immunotherapy [22], but the prognosis and prediction of CRGs in HCC have not been investigated. Correlation analysis showed that most of the CRGs were positively correlated with each other. The PPI network showed that LIAS, DLAT, PDHA1, PDHB, LIPT1, DLD were the central genes. In addition, according to GO enrichment analysis, 12 CRGs were mainly closely related to pyruvate dehydrogenase activity, oxidoreductase activity, mitochondrial pyruvate dehydrogenase complex and mitochondrial matrix. KEGG pathway analysis revealed that 12 CRGs were enriched in Citrate cycle (TCA cycle), Platinum drug resistance and Biosynthesis of cofactors. Many of these TCA pathways have been shown to be closely related to tumors. Besides, we performed a comprehensive bioinformatics analysis of HCC patients and identified prognostic features comprising four cuproptosis-genes (CDKN2A, DLAT, GLS, and PDHA1). And our predictive model shows a good ability to predict the prognosis of HCC patients. We analyzed the correlation between these four CRGs and immune processes in HCC patients. We also investigated the CRGs that correlated with pathological stages in HCC and developed five ceRNA networks to regulate their occurrence and progression. The immunohistochemical images of CDKN2A, DLAT, and PDHA1 in both HCC tissues and corresponding normal tissues confirmed the expression of the CRGs in HCC. Finally, the qRT-PCR experiments validated the expressions of CDKN2A, DLAT, GLS, and PDHA1 in LO2, 7791 and HepG2 cell lines.

To our knowledge, few reports on cuproptosis and HCC focus on the value and role of FDX15 [23]. However, the relationship between CRGs and HCC is still unclear. The roles of most CRGs in diseases such as esophageal cancer [24], endometrial cancer [25], and melanoma [10] are increasingly emerging. The prognostic score constructed in this study is based on four CRGs (CDKN2A, DLAT, GLS, and PDHA1). Further prognosis showed that OS with high levels of CRGs was worse. DLAT and PDHA1 are anti-cuproptosis genes, and GLS and CDKN2A sensitize cells to cuproptosis. These four genes were constructed as the CRGs most closely related to HCC in this paper.

DLAT is the initiator of the TCA cycle, and overexpressed DLAT promotes glycolysis and inhibits the breakdown of pyruvate into acetyl-CoA instead of promoting the TCA cycle, thereby aggravating the malignant development of NSCLC [26]; PDHA1 plays a role in HCC cancer metastasis and clinical pathology. The low expression of PDHA1 protein is associated with poor clinical outcomes in HCC patients [27], and the serine site of PDHA1, an important regulatory site in PDHC, has the most mutations in its subunits. Inhibition of PDHC reduces liver cell damage as a therapeutic target for liver failure [28]. Cancer cells can activate multiple glutamine metabolic pathways, and oncogenes control cancer malignant transformation by regulating glutamine uptake and metabolism. For example, overexpression of GLS2 greatly reduces tumor cell proliferation and cell colony formation [29], and GLS2 was confirmed as a tumor suppressor in HCC that inhibits tumor function by regulating glutamine metabolism [30]. As a tumor suppressor gene, CDKN2A encodes the p16 protein, which can inhibit the cell cycle, and up-regulation of CDKN2A can promote tumor proliferation [31]. All these indicate that CRGs are involved in the occurrence, development and prognosis of cancer.

In order to further study the mechanism of cuproptosis in HCC and help to establish more efficient immunotherapy strategies, we analyzed the relationship between CRGs and tumor microenvironment and immune infiltration. The results showed that CRGs had a good positive correlation with the abundance of various types of immune cells, and the level of immune cell infiltration was correlated with GLS and PDHA1 gene copy numbers in HCC. TMB was positively correlated with CDKN2A and negatively correlated with GLS; MSI was positively correlated with PDHA1 only. Therefore, we believe that CRGs may regulate the tumor immune microenvironment through multiple pathways rather than specifically targeting certain immune cells. Considering that immune cell infiltration is mainly regulated by various immunoinhibitors and immunostimulators [32], we evaluated the association of CRGs with immunoinhibitors and immunostimulators, and the results showed that CRGs were significantly associated with most immunoinhibitors and immunostimulators, indicating that CRGs are involved in potential role in immunotherapy.

In addition, a major finding of this study is that we found that five regulatory axes of CYTOR/MIR4435/DANCR-hsa-miR-125b-5p-CDKN2A, CYTOR/MIR4435-hsa-miR-125b-5p-GLS may be involved in the progression of HCC. Among them, hsa-miR-125b-5p plays an important role in the prognostic value of human early-stage lung cancer [33] and cervical cancer [34]. A previous study found that circulating hsa-miR-125b-5p was associated with HBsAg titers in patients with chronic HBV infection and regulated HBsAg expression [35]. Recent studies have found that hsa-miR-125b-5p is identified as playing a central role in controlling intestinal epithelial barrier function by targeting proteins involved in epithelial barrier function-related pathways, such as epithelial cell apoptosis, TJ, and actin-cytoskeleton signaling [36]. Hsa-miR-125b-5p regulates MMP-13 expression/production and several proinflammatory cytokines, chemokines or growth factors through TRAF6 mediated MAPKs and NF-κB signaling in stimulated human OA chondrocytes [37]. These findings suggest that hsa-miR-125b-5p plays an important regulatory role in various biological processes in humans, however, the specific molecular mechanism of hsa-miR-125b-5p remains to be studied. The five regulatory axes identified in this paper provide more theoretical basis for further research on the role of hsa-miR-125b-5p in human diseases.

Our research has multiple strengths. In this study, based on the characteristics of CRGs, a prognostic model of survival with HCC was constructed for the first time. Furthermore, the described mechanistic signaling is copper-dependent, which heralds a broader role for metal ions in cancer. However, this study still has shortcomings. Although these four genes showed strong prognostic prediction effects, sufficient data sets and clinical prognostic information are still needed for further validation in the future. Although CRGs showed good performance in predicting prognosis, some other genes with predictive value were not considered, such as why the gene with clear value FDX1 did not highlight its role in this study. Third, the statistical data constructed based on the database still needs further biological basis, such as *in vitro* and *in vivo* experiments.

## CONCLUSIONS

To date, we explored the characteristics of 12 CRGs in HCC tissue and predicted their association with survival, constructed a novel cuproptosis-related prognostic signature, and validated it by experiments. CRGs may be potential immunotherapy targets for HCC, and this study constructed five ceRNA networks with has-miR-125b-5p as the core, and the low expression of has-miR-125b-5p was confirmed to be an important regulator of poor prognosis miRNAs.

## Supplementary Material

Supplementary Figure 1

Supplementary Tables

## References

[1] Sung H, Ferlay J, Siegel RL, Laversanne M, Soerjomataram I, Jemal A, Bray F. Global Cancer Statistics 2020: GLOBOCAN Estimates of Incidence and Mortality Worldwide for 36 Cancers in 185 Countries. CA Cancer J Clin. 2021; 71:209–49. 10.3322/caac.21660 33538338

[2] Bai Y, Chen D, Cheng C, Li Z, Chi H, Zhang Y, Zhang X, Tang S, Zhao Q, Ang B, Zhang Y. Immunosuppressive landscape in hepatocellular carcinoma revealed by single-cell sequencing. Front Immunol. 2022; 13:950536. 10.3389/fimmu.2022.950536 35967424 PMC9365996

[3] Hartke J, Johnson M, Ghabril M. The diagnosis and treatment of hepatocellular carcinoma. Semin Diagn Pathol. 2017; 34:153–9. 10.1053/j.semdp.2016.12.011 28108047

[4] Bruix J, Llovet JM. Prognostic prediction and treatment strategy in hepatocellular carcinoma. Hepatology. 2002; 35:519–24. 10.1053/jhep.2002.32089 11870363

[5] Li X, Xu W, Kang W, Wong SH, Wang M, Zhou Y, Fang X, Zhang X, Yang H, Wong CH, To KF, Chan SL, Chan MTV, et al. Genomic analysis of liver cancer unveils novel driver genes and distinct prognostic features. Theranostics. 2018; 8:1740–51. 10.7150/thno.22010 29556353 PMC5858179

[6] Ji ZH, Ren WZ, Wang HQ, Gao W, Yuan B. Molecular Subtyping Based on Cuproptosis-Related Genes and Characterization of Tumor Microenvironment Infiltration in Kidney Renal Clear Cell Carcinoma. Front Oncol. 2022; 12:919083. 10.3389/fonc.2022.919083 35875087 PMC9299088

[7] Kahlson MA, Dixon SJ. Copper-induced cell death. Science. 2022; 375:1231–2. 10.1126/science.abo3959 35298241

[8] Tsvetkov P, Coy S, Petrova B, Dreishpoon M, Verma A, Abdusamad M, Rossen J, Joesch-Cohen L, Humeidi R, Spangler RD, Eaton JK, Frenkel E, Kocak M, et al. Copper induces cell death by targeting lipoylated TCA cycle proteins. Science. 2022; 375:1254–61. 10.1126/science.abf0529 35298263 PMC9273333

[9] Bian Z, Fan R, Xie L. A Novel Cuproptosis-Related Prognostic Gene Signature and Validation of Differential Expression in Clear Cell Renal Cell Carcinoma. Genes (Basel). 2022; 13:851. 10.3390/genes13050851 35627236 PMC9141858

[10] Lv H, Liu X, Zeng X, Liu Y, Zhang C, Zhang Q, Xu J. Comprehensive Analysis of Cuproptosis-Related Genes in Immune Infiltration and Prognosis in Melanoma. Front Pharmacol. 2022; 13:930041. 10.3389/fphar.2022.930041 35837286 PMC9273972

[11] Chen Y. Identification and Validation of Cuproptosis-Related Prognostic Signature and Associated Regulatory Axis in Uterine Corpus Endometrial Carcinoma. Front Genet. 2022; 13:912037. 10.3389/fgene.2022.912037 35937995 PMC9353190

[12] Jiang B, Zhu H, Feng W, Wan Z, Qi X, He R, Xie L, Li Y. Database Mining Detected a Cuproptosis-Related Prognostic Signature and a Related Regulatory Axis in Breast Cancer. Dis Markers. 2022; 2022:9004830. 10.1155/2022/9004830 36312586 PMC9605827

[13] Davis CI, Gu X, Kiefer RM, Ralle M, Gade TP, Brady DC. Altered copper homeostasis underlies sensitivity of hepatocellular carcinoma to copper chelation. Metallomics. 2020; 12:1995–2008. 10.1039/d0mt00156b 33146201 PMC8315290

[14] Li T, Fan J, Wang B, Traugh N, Chen Q, Liu JS, Li B, Liu XS. TIMER: A Web Server for Comprehensive Analysis of Tumor-Infiltrating Immune Cells. Cancer Res. 2017; 77:e108–10. 10.1158/0008-5472.CAN-17-0307 29092952 PMC6042652

[15] Asplund A, Edqvist PH, Schwenk JM, Pontén F. Antibodies for profiling the human proteome-The Human Protein Atlas as a resource for cancer research. Proteomics. 2012; 12:2067–77. 10.1002/pmic.201100504 22623277

[16] Yamamoto H, Watanabe Y, Maehata T, Imai K, Itoh F. Microsatellite instability in cancer: a novel landscape for diagnostic and therapeutic approach. Arch Toxicol. 2020; 94:3349–57. 10.1007/s00204-020-02833-z 32632538

[17] Samstein RM, Lee CH, Shoushtari AN, Hellmann MD, Shen R, Janjigian YY, Barron DA, Zehir A, Jordan EJ, Omuro A, Kaley TJ, Kendall SM, Motzer RJ, et al. Tumor mutational load predicts survival after immunotherapy across multiple cancer types. Nat Genet. 2019; 51:202–6. 10.1038/s41588-018-0312-8 30643254 PMC6365097

[18] Poznański J, Sołdacki D, Czarkowska-Pączek B, Bonna A, Kornasiewicz O, Krawczyk M, Bal W, Pączek L. Cirrhotic Liver of Liver Transplant Recipients Accumulate Silver and Co-Accumulate Copper. Int J Mol Sci. 2021; 22:1782. 10.3390/ijms22041782 33670100 PMC7916850

[19] Saleh SAK, Adly HM, Abdelkhaliq AA, Nassir AM. Serum Levels of Selenium, Zinc, Copper, Manganese, and Iron in Prostate Cancer Patients. Curr Urol. 2020; 14:44–9. 10.1159/000499261 32398996 PMC7206590

[20] Basu S, Singh MK, Singh TB, Bhartiya SK, Singh SP, Shukla VK. Heavy and trace metals in carcinoma of the gallbladder. World J Surg. 2013; 37:2641–6. 10.1007/s00268-013-2164-9 23942528

[21] Ge EJ, Bush AI, Casini A, Cobine PA, Cross JR, DeNicola GM, Dou QP, Franz KJ, Gohil VM, Gupta S, Kaler SG, Lutsenko S, Mittal V, et al. Connecting copper and cancer: from transition metal signalling to metalloplasia. Nat Rev Cancer. 2022; 22:102–13. 10.1038/s41568-021-00417-2 34764459 PMC8810673

[22] Zhang G, Sun J, Zhang X. A novel Cuproptosis-related LncRNA signature to predict prognosis in hepatocellular carcinoma. Sci Rep. 2022; 12:11325. 10.1038/s41598-022-15251-1 35790864 PMC9256635

[23] Zhang C, Zeng Y, Guo X, Shen H, Zhang J, Wang K, Ji M, Huang S. Pan-cancer analyses confirmed the cuproptosis-related gene FDX1 as an immunotherapy predictor and prognostic biomarker. Front Genet. 2022; 13:923737. 10.3389/fgene.2022.923737 35991547 PMC9388757

[24] Jiang R, Huan Y, Li Y, Gao X, Sun Q, Zhang F, Jiang T. Transcriptional and genetic alterations of cuproptosis-related genes correlated to malignancy and immune-infiltrate of esophageal carcinoma. Cell Death Discov. 2022; 8:370. 10.1038/s41420-022-01164-5 35995782 PMC9395517

[25] Shan J, Geng R, Zhang Y, Wei J, Liu J, Bai J. Identification of cuproptosis-related subtypes, establishment of a prognostic model and tumor immune landscape in endometrial carcinoma. Comput Biol Med. 2022; 149:105988. 10.1016/j.compbiomed.2022.105988 36007289

[26] Chen Q, Wang Y, Yang L, Sun L, Wen Y, Huang Y, Gao K, Yang W, Bai F, Ling L, Zhou Z, Zhang X, Xiong J, Zhai R. PM2.5 promotes NSCLC carcinogenesis through translationally and transcriptionally activating DLAT-mediated glycolysis reprograming. J Exp Clin Cancer Res. 2022; 41:229. 10.1186/s13046-022-02437-8 35869499 PMC9308224

[27] Sun J, Li J, Guo Z, Sun L, Juan C, Zhou Y, Gu H, Yu Y, Hu Q, Kan Q, Yu Z. Overexpression of Pyruvate Dehydrogenase E1α Subunit Inhibits Warburg Effect and Induces Cell Apoptosis Through Mitochondria-Mediated Pathway in Hepatocellular Carcinoma. Oncol Res. 2019; 27:407–14. 10.3727/096504018X15180451872087 29444744 PMC7848459

[28] Ferriero R, Nusco E, De Cegli R, Carissimo A, Manco G, Brunetti-Pierri N. Pyruvate dehydrogenase complex and lactate dehydrogenase are targets for therapy of acute liver failure. J Hepatol. 2018; 69:325–35. 10.1016/j.jhep.2018.03.016 29580866 PMC6057136

[29] Halama A, Suhre K. Advancing Cancer Treatment by Targeting Glutamine Metabolism-A Roadmap. Cancers (Basel). 2022; 14:553. 10.3390/cancers14030553 35158820 PMC8833671

[30] Suzuki S, Venkatesh D, Kanda H, Nakayama A, Hosokawa H, Lee E, Miki T, Stockwell BR, Yokote K, Tanaka T, Prives C. GLS2 Is a Tumor Suppressor and a Regulator of Ferroptosis in Hepatocellular Carcinoma. Cancer Res. 2022; 82:3209–22. 10.1158/0008-5472.CAN-21-3914 35895807 PMC11057045

[31] Yang L, Chen Y, Liu N, Lu Y, Ma W, Yang Z, Gan W, Li D. CircMET promotes tumor proliferation by enhancing CDKN2A mRNA decay and upregulating SMAD3. Mol Cancer. 2022; 21:23. 10.1186/s12943-022-01497-w 35042525 PMC8764797

[32] Chen D, Zhang X, Li Z, Zhu B. Metabolic regulatory crosstalk between tumor microenvironment and tumor-associated macrophages. Theranostics. 2021; 11:1016–30. 10.7150/thno.51777 33391518 PMC7738889

[33] Zeybek A, Öz N, Kalemci S, Edgünlü T, Kızıltuğ MT, Tosun K, Tunç M, Tekin L, Erdal ME. Diagnostic Value of MiR-125b as a Potential Biomarker for Stage I Lung Adenocarcinoma. Curr Mol Med. 2019; 19:216–27. 10.2174/1566524019666190314113800 30868951

[34] Wang T, Zhang XD, Hua KQ. A ceRNA network of BBOX1-AS1-hsa-miR-125b-5p/hsa-miR-125a-5p-CDKN2A shows prognostic value in cervical cancer. Taiwan J Obstet Gynecol. 2021; 60:253–61. 10.1016/j.tjog.2020.12.006 33678324

[35] Ninomiya M, Kondo Y, Kimura O, Funayama R, Nagashima T, Kogure T, Morosawa T, Tanaka Y, Nakayama K, Shimosegawa T. The expression of miR-125b-5p is increased in the serum of patients with chronic hepatitis B infection and inhibits the detection of hepatitis B virus surface antigen. J Viral Hepat. 2016; 23:330–9. 10.1111/jvh.12522 26924666

[36] Martínez C, Rodiño-Janeiro BK, Lobo B, Stanifer ML, Klaus B, Granzow M, González-Castro AM, Salvo-Romero E, Alonso-Cotoner C, Pigrau M, Roeth R, Rappold G, Huber W, et al. miR-16 and miR-125b are involved in barrier function dysregulation through the modulation of claudin-2 and cingulin expression in the jejunum in IBS with diarrhoea. Gut. 2017; 66:1537–8. 10.1136/gutjnl-2016-311477 28082316 PMC5561373

[37] Rasheed Z, Rasheed N, Abdulmonem WA, Khan MI. MicroRNA-125b-5p regulates IL-1β induced inflammatory genes via targeting TRAF6-mediated MAPKs and NF-κB signaling in human osteoarthritic chondrocytes. Sci Rep. 2019; 9:6882. 10.1038/s41598-019-42601-3 31053727 PMC6499837

