# Supplementary material for: Crosstalk of cuproptosis-related prognostic signature and competing endogenous RNAs regulation in hepatocellular carcinoma
Source: Aging (Albany NY). 2023 Dec 10;15(23):13901–19. doi: 10.18632/aging.205273 (PMC10756090; doi:10.18632/aging.205273)
Supplement: Supplementary Figure 1 [file aging-15-205273-s001.pdf]

SUPPLEMENTARY FIGURE

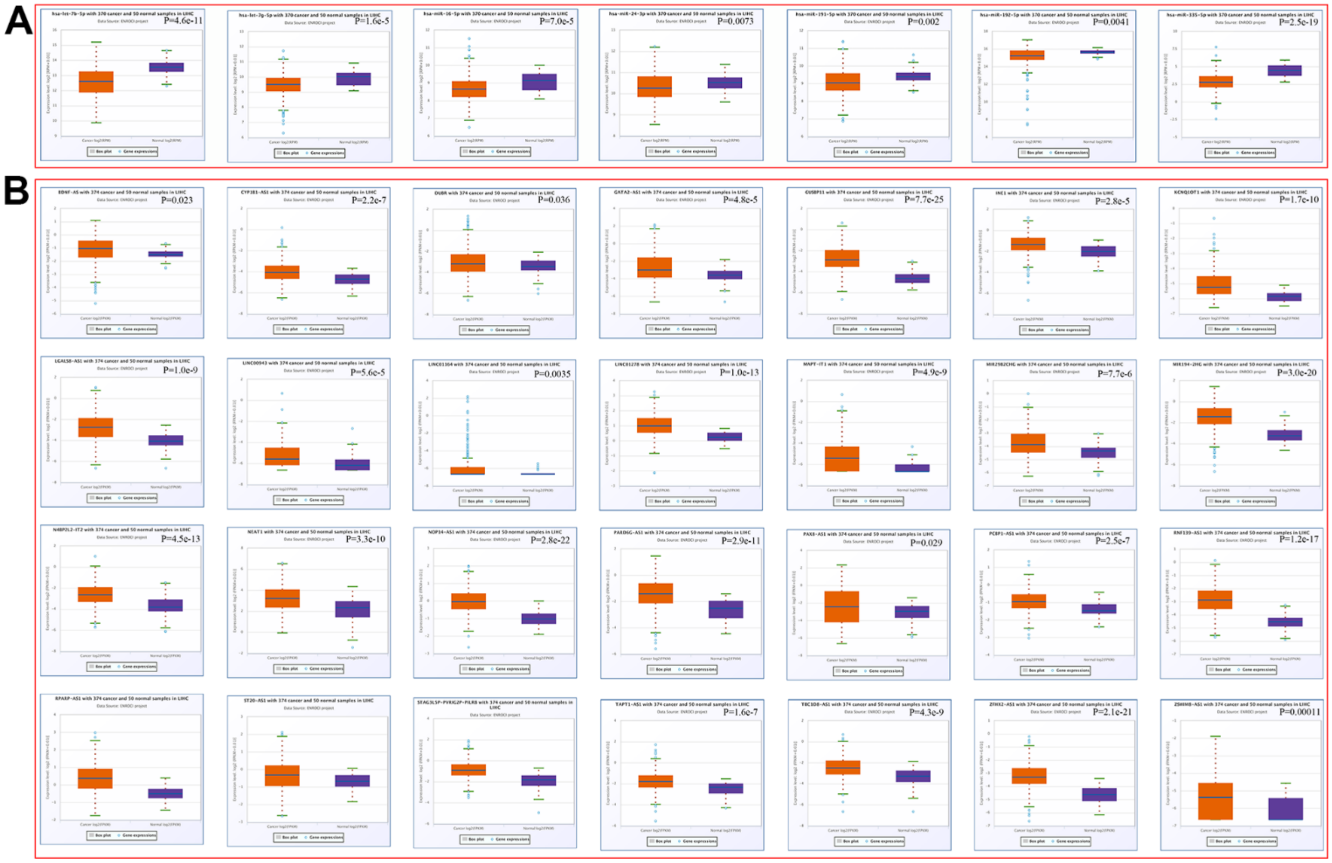

Supplementary Figure 1. The expression of upstream miRNAs and LncRNAs in HCC. (**A**) Down-regulated miRNAs. (**B**) Up-regulated LncRNAs.
