# Supplementary material for: Crosstalk of cuproptosis-related prognostic signature and competing endogenous RNAs regulation in hepatocellular carcinoma
Source: Aging (Albany NY). 2023 Dec 10;15(23):13901–19. doi: 10.18632/aging.205273 (PMC10756090; doi:10.18632/aging.205273)
Supplement: Supplementary Tables [file aging-15-205273-s002.pdf]

SUPPLEMENTARY TABLES

Supplementary Table 1. The mRNA-miRNA pairs predicted by mirTarBase and TarBase V.8 database.

mRNA	miRNA
GLS	hsa-miR-125b-5p
CDKN2A	hsa-miR-125b-5p
CDKN2A	hsa-miR-10b-5p
PDHA1	hsa-miR-10b-5p
DLAT	hsa-miR-10b-5p
CDKN2A	hsa-miR-24-3p
GLS	hsa-miR-24-3p
PDHA1	hsa-miR-24-3p
GLS	hsa-let-7b-5p
DLAT	hsa-let-7b-5p
CDKN2A	hsa-let-7b-5p
GLS	hsa-miR-34a-5p
CDKN2A	hsa-miR-34a-5p
GLS	hsa-miR-335-5p
GLS	hsa-miR-320a
CDKN2A	hsa-miR-320a
DLAT	hsa-miR-615-3p
CDKN2A	hsa-miR-615-3p
DLAT	hsa-miR-338-5p
PDHA1	hsa-miR-192-5p
DLAT	hsa-miR-192-5p
CDKN2A	hsa-miR-192-5p
PDHA1	hsa-miR-548k
GLS	hsa-miR-7-5p
GLS	hsa-miR-590-5p
GLS	hsa-let-7g-5p
DLAT	hsa-let-7g-5p
CDKN2A	hsa-let-7g-5p
PDHA1	hsa-miR-877-3p
GLS	hsa-miR-124-3p
CDKN2A	hsa-miR-124-3p
DLAT	hsa-miR-124-3p
GLS	hsa-miR-155-5p
CDKN2A	hsa-miR-155-5p
DLAT	hsa-miR-155-5p
GLS	hsa-miR-16-5p
CDKN2A	hsa-miR-16-5p
DLAT	hsa-miR-16-5p
CDKN2A	hsa-miR-191-5p
PDHA1	hsa-miR-191-5p

Supplementary Table 2. The miRNA-lncRNA pairs predicted by miRNet database.

miRNA	lncRNA
hsa-mir-125b-5p	LINC01128
hsa-mir-125b-5p	LINC01654
hsa-mir-125b-5p	MIR29B2CHG
hsa-mir-125b-5p	LGALS8-AS1
hsa-mir-125b-5p	CYP1B1-AS1
hsa-mir-125b-5p	PCBP1-AS1
hsa-mir-125b-5p	CYTOR
hsa-mir-125b-5p	TBC1D8-AS1
hsa-mir-125b-5p	MIR4435-2HG
hsa-mir-125b-5p	PAX8-AS1
hsa-mir-125b-5p	KLF7-IT1
hsa-mir-125b-5p	ACVR2B-AS1
hsa-mir-125b-5p	DUBR
hsa-mir-125b-5p	GATA2-AS1
hsa-mir-125b-5p	TNK2-AS1
hsa-mir-125b-5p	NOP14-AS1
hsa-mir-125b-5p	TAPT1-AS1
hsa-mir-125b-5p	DANCR
hsa-mir-125b-5p	STAG3L5P-PVRIG2P-PILRB
hsa-mir-125b-5p	LNCPRESS1
hsa-mir-125b-5p	RNF139-AS1
hsa-mir-125b-5p	GLIDR
hsa-mir-125b-5p	FAM27C
hsa-mir-125b-5p	ZSWIM8-AS1
hsa-mir-125b-5p	RPARP-AS1
hsa-mir-125b-5p	LINC01164
hsa-mir-125b-5p	KCNQ1OT1
hsa-mir-125b-5p	BDNF-AS
hsa-mir-125b-5p	MIR194-2HG
hsa-mir-125b-5p	NEAT1
hsa-mir-125b-5p	LINC00943
hsa-mir-125b-5p	N4BP2L2-IT2
hsa-mir-125b-5p	ZFHX2-AS1
hsa-mir-125b-5p	ST20-AS1
hsa-mir-125b-5p	LINC00273
hsa-mir-125b-5p	MAPT-IT1
hsa-mir-125b-5p	LINC00667
hsa-mir-125b-5p	PCAT18
hsa-mir-125b-5p	PARD6G-AS1
hsa-mir-125b-5p	LINC00661
hsa-mir-125b-5p	LINC00261
hsa-mir-125b-5p	PLAC4
hsa-mir-125b-5p	PICSAR
hsa-mir-125b-5p	GUSBP11
hsa-mir-125b-5p	INE1
hsa-mir-125b-5p	LINC01278
hsa-mir-125b-5p	XIST
